# Gut microbiota and physiologic bowel ^18^F-FDG uptake

**DOI:** 10.1186/s13550-017-0318-8

**Published:** 2017-08-31

**Authors:** Ji Yeon Kang, Han-Na Kim, Yoosoo Chang, Yeojun Yun, Seungho Ryu, Hocheol Shin, Hyung-Lae Kim

**Affiliations:** 1 Department of Nuclear Medicine, Kangbuk Samsung Hospital, Sungkyunkwan University School of Medicine, Seoul, South Korea; 2 Department of Biochemistry, Ewha Womans University, School of Medicine, 1071, Anyangcheon-ro, Yangcheon-gu, Seoul, 07985 South Korea; 3 Department of Occupational and Environmental Medicine, Kangbuk Samsung Hospital, Sungkyunkwan University School of Medicine, Seoul, South Korea; 4 Department of Family Medicine, Kangbuk Samsung Hospital, Sungkyunkwan University School of Medicine, 29, Saemunan-ro, Jongnogu, Seoul, 03181 South Korea

**Keywords:** ^18^F-FDG PET, Gut microbiota, Physiologic, Intestinal, Permeability

## Abstract

**Background:**

We investigated the association between physiologic bowel FDG uptake and gut microbiota. FDG uptake in the normal large and small intestine is widely variable both in distribution and intensity. The etiology of physiologic bowel ^18^F-FDG activity remains unknown.

**Results:**

We included 63 healthy male subjects. After overnight fasting, blood samples and ^18^F-FDG PET/CT scans were taken. Fecal samples were collected, and gut microbiota were analyzed by 16S rRNA gene-pyrosequencing. The physiologic bowel FDG uptake was classified into three groups by visual assessment and measured using the maximum and mean standardized uptake value. We used the total bowel to liver uptake ratio (TBR_max_ and TBR_mean_). There was no significant difference in age, BMI, or lipid profiles between groups. To identify specific microbial taxa associated with the bowel FDG uptake while accounting for age and BMI, we performed a generalized linear model. At the genus level, the group with focal or intense FDG uptake in the intestine was associated with low abundance of unclassified Clostridiales. The group with intestinal FDG uptake lower than the liver was associated with high abundance of *Klebsiella*. TBR_max_ and TBR_mean_ were negatively associated with abundance of unclassified Enterobacteriaceae.

**Conclusion:**

We cautiously speculate that physiologic bowel FDG activity might be caused by an increase in intestinal permeability and may reflect an impaired barrier function in the intestine.

**Electronic supplementary material:**

The online version of this article (doi:10.1186/s13550-017-0318-8) contains supplementary material, which is available to authorized users.

## Background

^18^F-FDG PET/CT is a useful tool in the evaluation of colonic malignancy and inflammatory bowel disease. FDG uptake in the normal large and small intestine is widely variable both in distribution and intensity. Uncommonly, it appears as focal activity, which makes it difficult to discriminate between malignancy and normal bowel tissue. Further investigations may be needed to exclude the possibility of malignancy, and these require additional time and cost.

The etiology of bowel FDG activity without pathologic lesions, also called “physiologic bowel uptake or activity”, is not thoroughly understood. Some previous studies have attempted to identify the cause of physiologic bowel FDG activity. Kim et al. demonstrated that ^18^F-FDG was located in the intestinal lumen by measuring the FDG activity of stool samples [1]. Soyka et al. reported that bowel preparation using a senna-glycoside solution before ^18^F-FDG PET/CT increased physiologic FDG activity in colonic structures, except for the sigmoid and rectum [2]. They postulated that ^18^F-FDG secretion was the main cause of physiologic activity. Senna-glycoside increases intestinal secretary activity by activation of chloride channels in the bowel wall. Tohihara et al. reported that physiologic bowel FDG uptake was increased more at the delayed phase than at the early phase in dual-time-point PET/CT imaging [3]. They also suggested that FDG secretion from the bowel wall may be related to physiologic uptake.

Franquet et al. reported that physiologic bowel FDG uptake was suppressed by antibiotics, such as rifaximin [4]. They suggested that bacteria play a role in accumulating FDG and may ingest FDG that has migrated into the intestinal lumen via transcellular translocation. This suggestion alone cannot account for individual differences in physiologic bowel FDG uptake. We hypothesized that the variability of intestinal FDG uptake may depend on a specific type of bacteria in the lumen. The relationship between intestinal FDG uptake and gut microbiota was investigated using high-throughput sequencing of the 16S rRNA gene in healthy male subjects.

## Methods

### Subjects

Participants were recruited from the Kangbuk Samsung Health Study, which is a cohort study of Korean men and women who undergo a comprehensive annual or biennial examination at Kangbuk Samsung Hospital Screening Centers in South Korea. Stool samples were collected from 1463 adult participants (men: 907, women: 556) between the ages of 23 and 78 who underwent a comprehensive health checkup between June 2014 and September 2014. Among them, 76 males and 5 females had completed PET images and only males were included in this study.

### Inclusion and exclusion criteria

Participants who met any of the exclusion criteria were not enrolled in this study. We excluded one participant with less than 5000 sequences per sample and five participants with antibiotic use within 6 weeks prior to enrollment, regular use of statins, or probiotic use within 4 weeks prior to enrollment. To avoid bowel FDG uptake caused by Metformin, we excluded six participants who had diabetes, were taking a medication for diabetes, or had a fasting glucose level ≥ 126 mg/dL. We excluded a participant showing a hot spot at the left femur in an ^18^F-FDG PET/CT image, which is suspicious for a benign or malignant tumor. Finally, 63 participants (100% male) with a mean of 25,077 sequences per sample were included.

### Measurements

Medical history was collected through a self-administered questionnaire. Body mass index (BMI) was calculated as weight in kilograms divided by height in meters squared. After fasting overnight, blood samples were taken from the antecubital vein before the ^18^F-FDG PET/CT scan. Serum glucose, HbA1c, total cholesterol, triglycerides, uric acid, high-density lipoprotein, low-density lipoprotein, high-sensitivity C-reactive protein, free T4, free T3, and TSH were measured according to standard procedures.

### PET/CT protocol

Participants were asked to fast overnight before PET/CT scan. Blood glucose levels at the time of injection of ^18^F-FDG were lower than 160 mg/dl. PET/CT scans were performed on Discovery D600 (for 57 subjects) or Discovery STE scanners (for 6 subjects) (GE Medical Systems, Waukesha, WI, USA) with a tracer uptake time of 60 min. ^18^F-FDG was injected based on weight (0.1 mCi/kg on the D600 scanner, 10-13 mCi on the STE scanner). No intravenous contrast agent was given. For the Discovery D600 scanner, slice thickness was 3.75 mm, current was 40–120 mAs, and energy was 120 kVp. For the Discovery STE scanner, slice thickness was 3.3 mm, current was 40–200 mAs, and energy was 140 kVp. Following CT acquisition, a PET emission scan was acquired with an acquisition time of 2.5–3 min per bed in 3D mode from the proximal thigh to the skull base. CT data were used for attenuation correction. The images were reconstructed using a conventional iterative algorithm (OSEM). Using dedicated software (Advanced Workstation, GE Healthcare, Milwaukee, WI, USA), CT, PET, and fused PET/CT images were reviewed.

### Image analysis

**Fig. 1:**
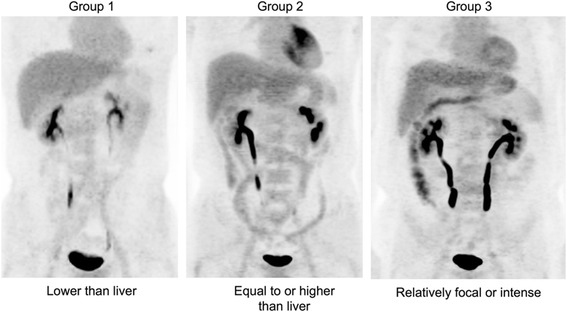
Intestinal ^18^F-FDG uptakes classified by visual analysis

We assessed intestinal ^18^F-FDG uptake by visual analysis and quantitative analysis. For the visual analysis, we classified subjects into three groups. Group 1 included subjects with intestinal FDG uptake lower than liver FDG uptake. Group 2 included subjects with intestinal FDG uptake equal to or higher than liver FDG uptake; among group 2 subjects, subjects with relatively focal or intense FDG uptake in the intestine were reclassified as group 3 (Fig. 1). For the quantitative analysis, we measured maximum and mean standardized uptake values (SUV_max_ and SUV_mean_) in each segment of the intestine using a three-dimensional volume of interest (VOI). The intestine was divided as follows: third portion of the duodenum, jejunum, ileal loop, ileocecal junction, ascending colon, transverse colon, descending colon, and sigmoid colon. The SUV_mean_ of each segment was measured with a margin threshold of 60% SUV_max_ [5]. After SUV measurement, we added all the segment values, which indicated the SUVs of the total bowel: TB SUV_max_ and TB SUV_mean_. For the measurement of liver SUV_mean_, we drew 3 cm VOIs in both lobes of the liver as previously reported [6]. The uptake ratio of TB SUV_max_ and TB SUV_mean_ to liver SUV_mean_ (TB to liver uptake ratio, TBR_max_ and TBR_mean_) was calculated for each subject.

### Fecal samples and DNA extraction

Fecal samples were immediately frozen after defecation at −20 °C and were placed at −70 °C within 24 h. Within 1 month, the stool specimens were vortexed to achieve a homogenous suspension and homogenized to isolate DNA using the PowerSoil® DNA Isolation Kit (MO BIO Laboratories, Carlsbad, CA) according to the manufacturer’s instructions.

### PCR amplification and sequencing of the 16S rRNA gene

Variable V3 and V4 regions of the 16S rRNA were amplified with the universal primers F319 (5′ TCGTCGGCAGCGTCAGATGTGTATAAGAGACAG) and R806 (5′–GGACTACHVGGGTWTCTAAT–3′) [7], with each primer modified to contain a unique 8-nt barcode index by combination with the NexteraR XT DNA Library Preparation kit (Illumina, San Diego, CA). PCR reactions contained 5 ng/uL of DNA template, 2× KAPA HiFi HotStart Ready Mix (KAPA Biosystems, Wilmington, MA), and 2 pmol of each primer. Reaction conditions consisted of an initial incubation at 95 °C for 3 min, followed by 25 cycles of 95 °C for 30 s, 55 °C for 30 s, and 72 °C for 30 s. Samples were subjected to a final extension incubation at 72 °C for 5 min. After PCR clean-up and index PCR, sequencing was performed on the Illumina MiSeq platform according to the manufacturer’s specifications [8, 9]. The 100 bp of overlapping paired-end reads were merged using PandaSeq (version 2.7). To analyze the 16S rRNA gene sequence, quality filtering, including determination of the sequence length (>300 bp), end trimming, and determination of the number of ambiguous bases and the minimum quality score were performed using Trimmomatic (version 0.32).

### Sequence analysis using QIIME

Chimeras were detected and removed using USERCH 6.1 within the QIIME package (version 1.9) [10]. To identify OTUs from the non-chimeric sequences, an open-reference OTU picking approach was performed using representative sequences with pre-assigned taxonomy from Greengenes (version 13_8) [11]. This analysis was performed in QIIME, with a 97% similarity threshold. In an open-reference OTU picking process, reads are clustered against a reference sequence collection, and any reads that do not hit the reference sequence collection are subsequently clustered de novo. The initial data set for all 1463 samples included 278,619 OTUs, and the sequencing depth ranged from 38 to 137,059 reads per sample (mean = 24,644, SD = 17,384). OTUs with <0.005% of the total number of sequences were discarded as recommended by Navas-Molina et al. [12] for downstream analysis, resulting in 1011 OTUs.

### Statistical analysis

For cross-sectional analyses of baseline characteristics, differences were evaluated using the Kruskal-Wallis test and the Spearman correlation test. STATA version 13.0 (StataCorp LP, College Station, Texas, USA) was used for these analyses. All statistical analyses were performed in RStudio (version 0.98.983) using packages phyloseq 1.14.0 [13], DESeq2 1.10.1 [14] and ggplot2 2.1.0 [15]. We compared the composition and structure of the gut microbiota between group 1 vs. 2, group 1 vs. 3, group 2 vs. 3, group 1 vs. 2 and 3, and groups 1 and 2 vs. 3. Various indices (Richness: Chao1, ACE; richness and evenness: Shannon, InvSimpson) were obtained using the estimate_richness function of the phyloseq package with untrimmed datasets for meaningful results, as described by the phyloseq manual. Statistical tests for alpha diversity among groups of ^18^F-FDG uptake were performed using the *t* test and Wilcoxon rank-sum test with the mentioned indices. Beta diversity was obtained using the ordinate function of the phyloseq package. For beta diversity analysis, we normalized OTU matrices with a cumulative sum scaling (CSS) approach [16]. To identify factors that could drive groupings of similar communities, principal coordinate analysis (PCoA) was performed on the Bray-Curtis matrices generated from beta diversity calculation, and the resulting PCoA plots were visualized using the phyloseq. The Bray–Curtis index of similarity was calculated to investigate the similarity of microbiota composition between different samples.

To determine statistically significant differences between samples from groups 1, 2, and 3, taxa seen more than three times in at least 10% of the samples were removed from the analyses to protect against an OTU with a small mean and a trivially large coefficient of variation. The number of OTU-mapped reads was normalized across all samples using the variance-stabilized transformation method as implemented in DESeq2 using a generalized linear model (GLM). Statistical inference was conducted using the negative binomial GLM fitting and Wald significance tests. We adjusted for age and BMI by including them as covariates in the linear model. The resulting *p* values were corrected for multiple comparisons at each phylogenetic level using the Benjamini-Hochberg correction (FDR). Significant findings were reported for OTUs that had fold changes exceeding two in any group and significant statistical associations (FDR *q* < 0.05).

## Results

### Participant characteristics

All the subjects had no history of cancer, diabetes, heart disease including coronary disease, stroke, or inflammatory bowel disease. Four of 63 subjects had a history of hypertension. Participant characteristics are summarized in Table 1. The mean age was 44.7 (range 39–58) years. Twenty-six subjects (41%) were in group 1, 20 (31%) were in group 2, and 17 (27%) were in group 3. There was no significant difference in age and body mass index (BMI) between groups. There were significant differences in HbA1c, C-reactive protein (CRP), and Free T4 between the groups. Serum glucose and lipid profiles showed no significant differences. Measured values for the quantitative analysis, TBR_max_ and TBR_mean_, showed strong significant differences between groups sorted by visual analysis. In the Spearman correlation test, TBR_max_ and TBR_mean_ were evaluated with age, BMI and laboratory data. There was no significant correlation between them.

**Table 1:** Demographic, laboratory and TBR data by visual grade of intestinal FDG uptake

	Group 1 (*n* = 26)	Group 2 (*n* = 20)	Group 3 (*n* = 17)	*p value*
Age (years)	43.8 ± 4.5	45.5 ± 3.8	45.2 ± 5.4	*0.17*
BMI (kg/m^2^)	24.8 ± 1.8	23.7 ± 2.4	25.2 ± 2.6	*0.09*
Fasting Glucose (mg/dl)	92.3 ± 9.3	90.8 ± 6.1	95.4 ± 6.8	*0.17*
HbA1c(%)	5.47 ± 0.18	5.36 ± 0.16	5.39 ± 0.19	0.04*
Cholesterol (mg/dl)	210.5 ± 31.2	189.9 ± 29.5	208 ± 38.3	*0.15*
Triglyceride (mg/dl)	131.8 ± 72.7	132.7 ± 94.2	150.8 ± 73.3	*0.28*
HDL (mg/dl)	53.9 ± 9.8	49.5 ± 11.3	52.9 ± 12.2	*0.44*
LDL (mg/dl)	134.4 ± 26.5	116.7 ± 25.6	130.2 ± 30.4	*0.08*
CRP (mg/dl)	0.065 ± 0.083	0.045 ± 0.031	0.129 ± 0.156	0.04*
Uric Acid (mg/dl)	5.77 ± 1.03	6.28 ± 0.96	6.61 ± 1.17	*0.12*
Free T4 (ng/dl)	1.31 ± 0.15	1.34 ± 0.11	1.18 ± 0.17	0.01*
Free T3 (pg/dl)	3.09 ± 0.32	3.21 ± 0.28	3.05 ± 0.25	*0.21*
TSH (uIU/ml)	1.59 ± 0.53	1.38 ± 0.7	1.94 ± 1.18	*0.15*
TBR_max_	16.2 ± 1.6	18.6 ± 1.8	20.9 ± 3.1	<0.001*
TBR_mean_	11.5 ± 1.6	13.5 ± 1.3	15.1 ± 2.3	<0.001*
Library size mean	22,331 ± 12,250	23,923 ± 13,767	30,636 ± 25,333	*0.29*

Data are mean ± standard deviation

*BMI* body mass index, *HDL* high-density lipoprotein, *LDL* low-density lipoprotein, *CRP* C-reactive protein, *TSH* thyroid-stimulating hormone

*p < 0.05

The 16S RNA gene sequencing produced 1,579,858 sequences across 63 samples. The average number of sequences was 22,331, 23,923, and 30,636 for groups 1, 2, and 3, respectively. The number of generated sequences was not affected by grouping for intestinal ^18^F -FDG uptake (*p* = 0.29).

### ^18^F -FDG uptake and alpha and beta diversity in the gut microbiome

Maintaining sufficient bacterial richness and diversity is important for providing gut microbiota with functional redundancy, adaptability, and thus systematic robustness against environmental challenges [17]. Using a total of 1,579,858 qualified sequences, we estimated bacterial richness and evenness. The Chao1 and ACE indices for alpha diversity were used to estimate the number of species (richness) in the microbiome, with correction for subsampling and metrics that aim to measure diversity by accounting for evenness or homogeneity using Shannon and InvSimpson. We compared the indices between group 1 vs. 2, group 1 vs. 3, group 2 vs. 3, group 1 vs. 2 and 3, and groups 1 and 2 vs. 3. There was no significant difference in ^18^F-FDG uptake in all indices of alpha diversity (Additional file 1: Figure S1 and Table S1) between groups.

The PERMANOVA analysis showed a significant (*p* = 0.047, pseudo-*F* = 1.57) difference in Bray-Curtis distances between groups 1 and 2, although the comparison of the bacterial communities by principal coordinate analysis (PCoA) plot with the Bray-Curtis distance could not easily distinguish between the two groups (Additional file 1: Figure S2).

### Association of intestinal ^18^F-FDG uptake with gut microbiota

**Fig. 2:**
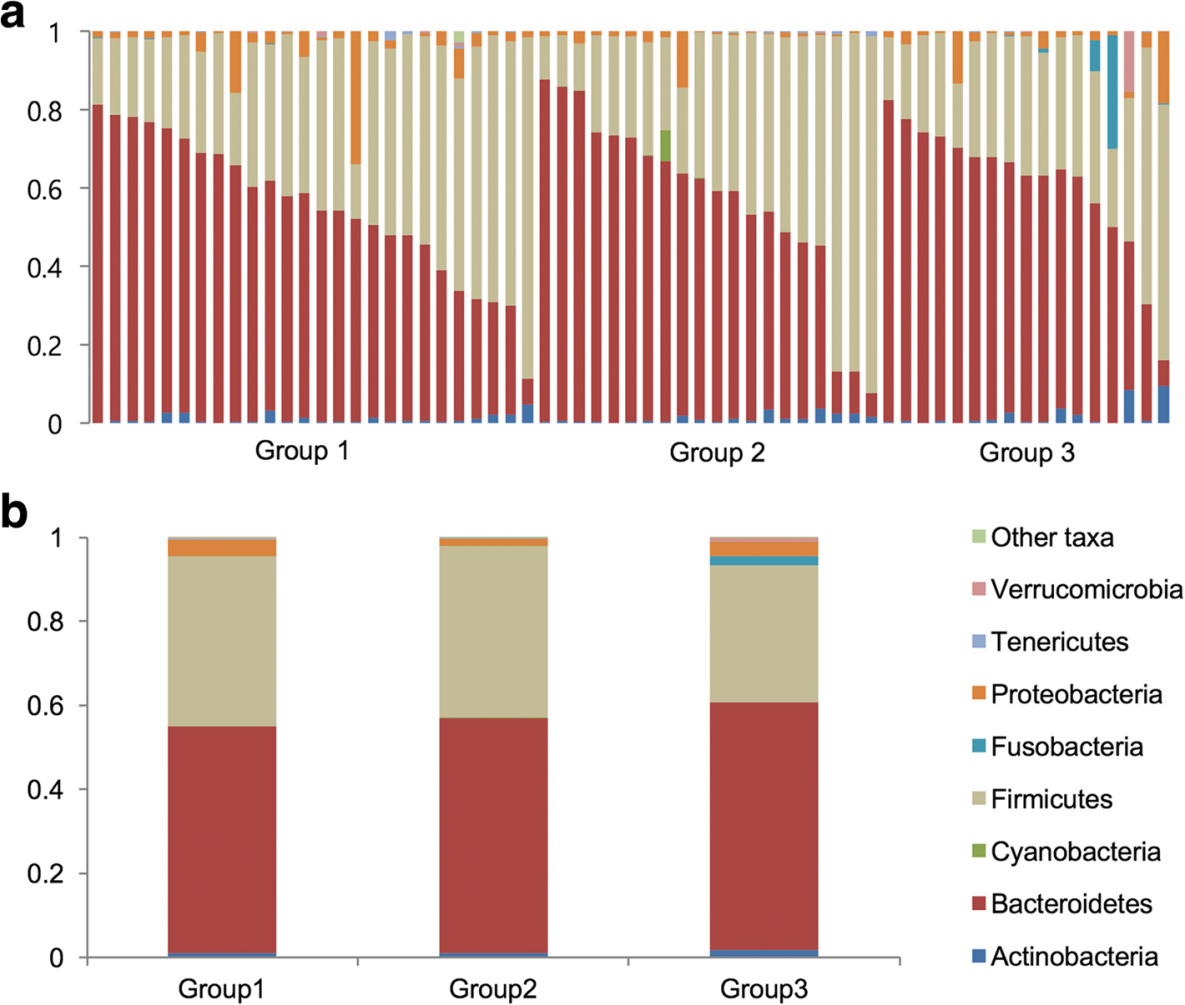
Relative abundances of microbial phyla in each subject (**a**) and group (**b**) (group 1; *n* = 26, group 2; *n* = 20, group 3; *n* = 17) (Most abundant eight phyla were demonstrated according to the phylum Bacteriodetes)

We next examined whether differences in the abundances of specific bacterial taxa were associated with intestinal ^18^F -FDG uptake. For detailed taxonomic analyses, individual sequences were classified and assigned to 11 known phyla. Firmicutes and Bacteroidetes were the two most dominant phyla and they comprised an average of 93.04% of total classifiable sequences in all groups (group 1: 92.77%, group 2: 93.95%, and group 3: 92.37%) (Fig. 2), as previously reported in many studies [18]. Comparison of the mean abundances between groups by the Student’s *t* test showed that the phylum Fusobacteria was more abundant in group 3 than in group 1 (*F* = 14.44, *p* < 0.0001). Since the abundance data were not normally distributed and contained a large fraction of zero values, we employed a negative binomial GLM using DESeq, which was used as the main statistical test for comparison throughout this study. The tests showed that Fusobacteria were significantly associated with differences between groups 1 and 3 [log_2_ fold change (log2FC) =4.50, *q* = 9.32 × 10^−3^] (Additional file 1: Table S2-2).

**Fig. 3:**
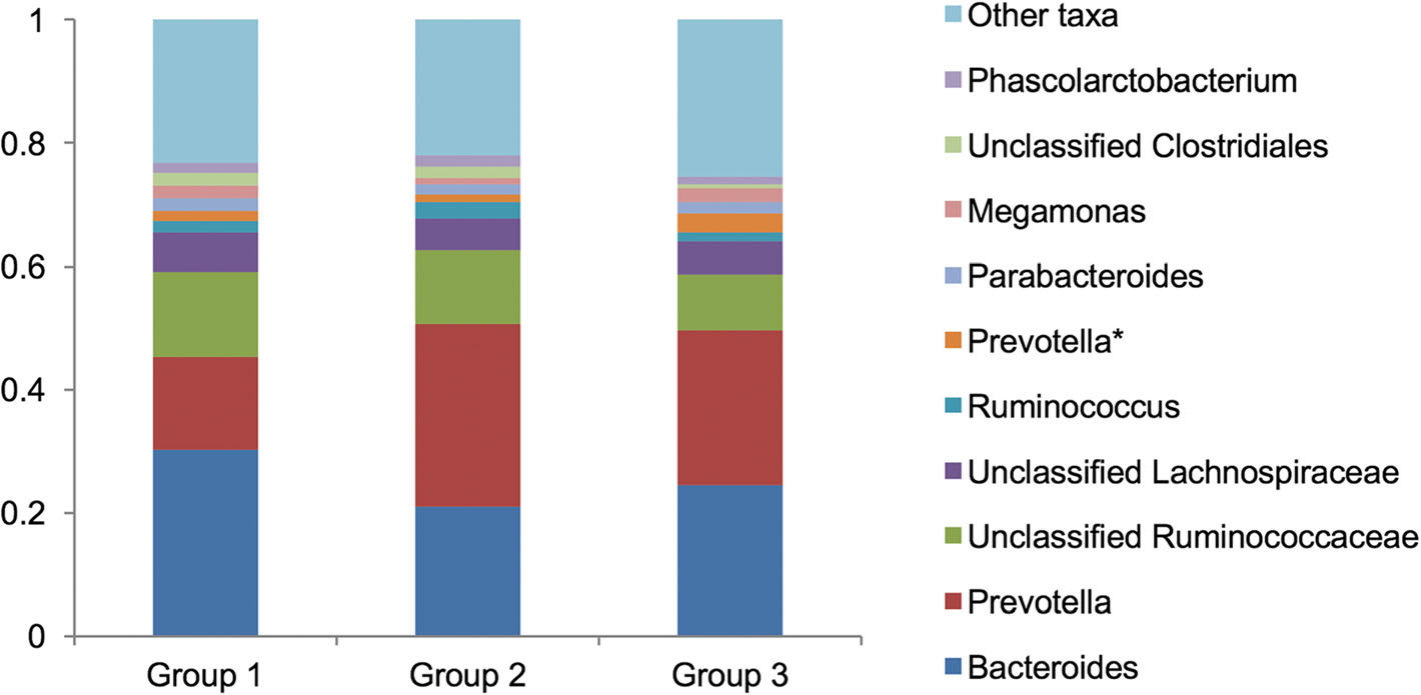
Relative abundances of the ten most genera in each group. ^*^ This genus *Prevotella* belongs to the family Paraprevotellaceae

**Fig. 4:**
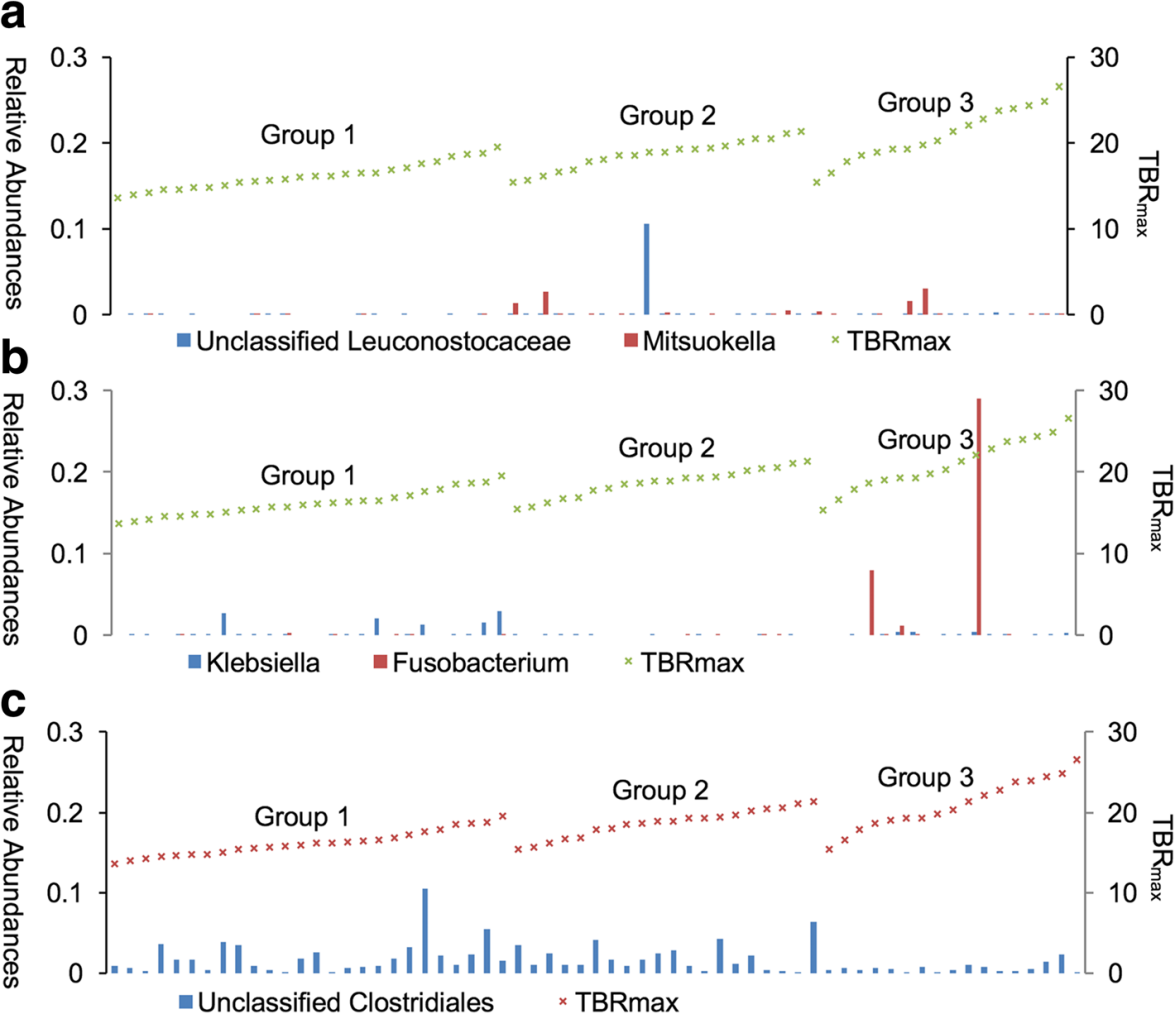
Relative abundances of the genera showing significant difference between groups; (**﻿a**)Unclassified Leuconostocaceaeand *Mitsuokella*, (**b**) *Klebsiella* and *Fusobacterium*, (**c**) unclassified clostridiales (Data are demonstrated according to TBR_max_)

At the genus level, among 88 genera identified, *Bacteroides spp.*, *Prevotella spp*., unclassified Ruminococcaceae, unclassified Lachnospiraceae, and *Ruminococcaceae spp*. were the top five most abundant genera identified (Fig. 3). The five genera comprised 56.52, 57.37, and 54.42% of groups 1, 2, and 3, respectively. The unclassified Leuconostocaceae and *Mitsuokella spp.* were significantly more abundant in group 2 than in group 1 after controlling for age and BMI (log2FC = 5.29, 4.46; *q* = 4.02 × 10^−7^, 2.85 × 10^−3^, respectively) (Table 2 and Fig. 4a). In contrast, the abundance of *Klebsiella spp.* was lower in group 2 than in group 1 (log2FC = −3.00, *q* = 1.81 × 10^−2^) (Fig. 4b). *Mitsuokella spp.* were also more abundant in group 3 than in group 1 (log2FC = 4.84, *q* = 3.85 × 10^−2^). We found that group 3 had more abundant *Fusobacterium spp.* than group 1 (log2FC = 5.74, *q* = 3.85 × 10^−2^), as well as the phylum Fusobacteria (Additional file 1: Table S2-2). However, we could not find the significant differences of taxa between group 2 and group 3 in the phylum and genus level (Additional file 1: Table S2-3 and Table S3). Moreover, there was no statistically significant difference in the unclassified Leuconostocaceae and *Fusobacterium* between group 2 and group 3 although there seemed to be differences in the Fig. 4.

**Table 2:** Differing abundance of specific populations of gut microbiota among groups for intestinal ^18^F-FDG uptake at the level of genus

Group	Genus	Relative Abundance (%)^a^	Log2FoldChange (SE)	*p value*	*q value* ^b^
1 vs. 2	Unclassified Leuconostocaceae	0.14	5.29 (0.91)	6.27 × 10^−9^	4.02 × 10^−7^
	*Mitsuokella*	0.24	4.46 (1.14)	8.91 × 10^−5^	2.85 × 10^−3^
	*Klebsiella*	0.36	−3.00 (0.90)	8.49 × 10^−4^	1.81 × 10^−2^
1 vs. 3	*Mitsuokella*	0.24	4.84 (1.26)	1.24 × 10^−4^	3.85 × 10^−2^
	*Fusobacterium*	0.20	5.74 (1.49)	1.19 × 10^−4^	3.85 × 10^−2^
1 vs. 2 + 3	Unclassified Leuconostocaceae	0.14	4.73 (0.81)	4.39 × 10^−9^	2.85 × 10^−7^
	*Mitsuokella*	0.24	4.35 (1.01)	1.73 × 10^−5^	5.62 × 10^−4^
	*Klebsiella*	0.36	−2.41 (0.76)	1.57 × 10^−3^	3.40 × 10^−2^
1 + 2 vs. 3	*Fusobacterium*	0.20	4.68 (1.07)	1.25 × 10^−5^	8.10 × 10^−4^
	Unclassified Clostridiales	4.10	−1.11 (0.31)	3.88 × 10^−4^	1.26 × 10^−2^
	Unclassified Leuconostocaceae	0.14	−2.83 (0.87)	1.17 × 10^−3^	2.53 × 10^−2^

The results of between group 2 and group 3 were not shown because there is no taxon with significant difference (*q* value <0.05)

^a^Relative abundance in total 63 samples

^b^ *q* value <0.05, the *q* values were calculated using Benjamini-Hochberg correction (FDR)

To compare subjects showing diffuse FDG activity and relatively intense FDG activity in the intestine, we combined groups 1 and 2 and compared them with group 3. To compare subjects with lower FDG activity and higher activity than the liver, we combined groups 2 and 3 and compared them with group 1. The unclassified Clostridiales were more abundant in the group 1 + 2 than group 3 (log2FC = −1.11, *q* = 1.26 × 10^−2^) (Fig. 4c). The abundance of unclassified Clostridiales was shown the negative trend by groups in Fig. 4c and the taxon was also more abundant in group 2 than group 3 although it was not statistical significant (*p* = 0.003, *q* = 0.18) in (Additional file 1: Table S3). Comparison between groups 1 and 2 + 3 yielded results similar to those obtained from the comparison of groups 1 and 2.

### Quantitative analysis *of intestinal*^*18*^*F-FDG uptake*

**Fig. 5:**
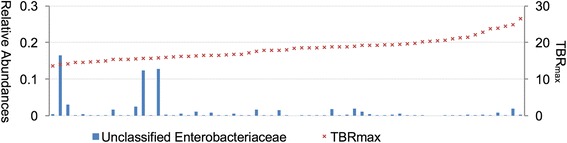
The association between the unclassified Enterobacteriaceae and TBR_max_

We also tested whether TBR_max_ and TBR_mean_ were associated with changes in gut microbiota. The TBR_max_ and TBR_mean_ showed similar results (Table 3). At the phylum level, there was no significant association between TBR and the abundance of individual phyla. At the genus level, abundance of the unclassified Enterobacteriaceae was negatively associated with TBR_max_ and TBR_mean_ (log2FC = −0.35, −0.47; *q* = 3.56 × 10^−3^, 4.38 × 10^−3^, respectively) (Fig. 5).

**Table 3:** Association gut microbiota and TBR_max_ and TBR_mean_ at the level of genus

	Genus	Relative abundance (%)^a^	Log2 foldchange (SE)	*p value*	*q value* ^b^
TBR_mean_	Unclassified Enterobacteriaceae	1.26	−0.47 (0.12)	6.75 × 10^−5^	4.38 × 10^−3^
	Unclassified Enterobacteriaceae	0.33	−0.47 (0.13)	2.60 × 10^−4^	8.44 × 10^−3^
TBR_max_	Unclassified Enterobacteriaceae	1.26	−0.35 (0.09)	5.48 × 10^−5^	3.56 × 10^−3^
	Unclassified Enterobacteriaceae	0.33	−0.34 (0.09)	3.45 × 10^−4^	1.12 × 10^−2^

^a^Relative abundance in total 63 samples

^b^ *q* value <0.05, *q*-values were calculated using Benjamini-Hochberg correction (FDR)

## Discussion

In this study, unclassified Clostridiales were consistently lower in group 3 than in the other groups. It was previously reported that unclassified Clostridiales were decreased in rats fed a high-fat diet (HFD) or the FODMAP (fermentable oligo-, di- and mono-saccharides and polyols) diet (S. Zhou, unpublished data, 2013). These diets caused dysbiosis in the gut accompanied by mucosal inflammation and impaired permeability. Another study reported that unclassified Clostridiales were inversely correlated with fat mass in rats fed a HFD and high protein diet (J. Jacobs, unpublished data, 2016). In a human study, Verdam et al. observed that obese subjects had lower uncultured Clostridiales than non-obese subjects [19].

The relationship between diet and bowel FDG uptake is still unclear; however, we can cautiously speculate regarding the associations between these factors. A previous study demonstrated that the SUV of background intestinal FDG uptake was positively correlated with BMI and lipid profile in female Korean patients with breast cancer [5]. However, we did not observe a significant difference in BMI and lipid profiles based on bowel FDG activity. Our study population contained only a small number of men with a young and narrow age distribution who were healthier than the population of the aforementioned study. Due to these dissimilarities, the difference in BMI and lipid profile among groups may not be noticeable.

HFD leads to intestinal inflammation, metabolic endotoxemia, increased intestinal permeability, and obesity [20, 21]. If physiologic bowel FDG uptake is associated with intestinal permeability, many factors are likely to affect bowel FDG activity, including the HFD. Obesity [22], stress [23], irritable bowel syndrome [24], and a variety of autoimmune diseases [25], all of which are known to increase intestinal permeability, may be associated with physiologic bowel FDG uptake.

A previously mentioned study [4] demonstrated suppressed bowel FDG uptake by Rifaximin and showed that bacteria are responsible for the retention of FDG. Accordingly, FDG activity is predicted to be determined by the number of bacteria. In our study, the *Fusobacterium* was more abundant in group 3 than in the other groups. However, 11 out of 17 subjects in group 3 were found not to have *Fusobacterium* and only three patients showed prominent abundance. These findings suggested that they might be incidental and not actually related to bowel FDG uptake.

In a study by Xu et al., water-avoidance stress in rats induced intestinal inflammation and increased intestinal permeability. They also improved intestinal permeability through chronic treatment with Rifaximin [26]. Rifaximin was reported to prevent stress-induced mucosal inflammation, intestinal barrier impairment, and visceral hyperalgesia, which was linked to enhanced gut permeability. It is possible that contrary to previous suggestions [4], suppressed intestinal FDG activity by rifaximin might be due to improved intestinal permeability rather than a bactericidal effect.

Group 1 had increased *Klebsiella* compared to group 2. The *Klebsiella* was also more abundant in group 1 than group 3 although it was not statistical significant (*p* = 0.06, *q* = 0.36). And the unclassified Enterobacteriaceae were negatively correlated with the TBR value in this study. We observed a prominent abundance of these genera in only a few subjects, so the possibility of incidental findings needs to be considered. These genera belong to the family *Enterobacteriaceae*. Their metabolic outcomes include enhancement of intestinal barrier integrity and reduction in the permeability of bacterial components [27]. Therefore, when physiologic bowel activity is scant, the intestinal barrier can be maintained by these bacteria.

It is unclear whether FDG translocates from the blood to the intestinal lumen through a transcellular pathway or paracellular pathway. If bowel FDG activity is related to intestinal permeability, FDG is likely to migrate through a paracellular pathway because intestinal permeability is regulated by paracellular tight junction [25]. Levine et al. showed a good correlation between intestinal glucose secretion and blood glucose concentration in rats [28]. They suggested that glucose moves bidirectionally across the intestine, and that its secretion is a passive process. Recently, Bardhan et al. postulated a paracellular route of FDG and refuted the hypothesis of transcellular transport of FDG [29]. Moreover, they refuted the transcellular hypothesis of FDG via the GLUT transporter suggested by Wang et al. [30]. The GLUT transporters located on the basolateral surface of the mucosal cells are known to export glucose from mucosal cells to the blood in the small intestine. Bardhan et al. suggested that it is doubtful that GLUT transporters also exist in the large bowel and speculated as to whether they can also transport in the opposite direction.

In our study, the relative abundance of the *Fusobacterium* was 0.2% and it is not an abundant taxon. The prominent abundance of Fusobacterium was observed in group 3, but not in the other groups. It might be possible that unusual bacteria proliferate in the intestinal lumen due to intestinal barrier dysfunction or a change in the microbial population. The potential of Fusobacterium seems to be worth describing although we could not exclude the possibility of an incidental association due to small sample size.

*Fusobacterium* was reported to be increased in obese people compared to lean people in a Japanese study population [31]. A majority of studies reported that the *Fusobacterium* was enriched in intestinal inflammation, colorectal adenoma, and cancer [32, 33]. Recently, a Korean study reported that the prevalence of colorectal adenoma was increased according to physiologic bowel FDG activity [34]. The authors assumed that the contribution of gut flora may explain the significant association between background intestinal ^18^F-FDG uptake and the prevalence of colorectal adenoma. *Fusobacterium* might explain these associations, but further investigation is needed to ascertain the link between the *Fusobacterium* and physiologic FDG uptake.

The associations between *Fusobacterium* and obesity and between obesity and physiologic bowel FDG uptake have not yet been revealed in the western population. It is well known that certain dietary habits influence the composition of gut microbiota. The aforementioned associations may have appeared prominently in Korean and Japanese subjects, which have relatively similar food cultures compared with western countries. Although multiculturalism has reduced lifestyle differences between Asian and Western populations, significant distinctions persist, including patterns of food consumption, multiple products of gut microbial and host co-metabolism, and gut microbiota compositions [35].

In this study, unclassified Leuconostocaceae was increased in group 2, but only one subject showed a prominent abundance. This finding is likely to be incidental and not related to FDG uptake.

This study has several limitations. First, there were a small number of subjects, and a larger cohort study is needed to ascertain whether an association truly exists or not. Second, we grouped subjects by visual analysis in the assessment of bowel FDG uptake. Visual analysis is subjective and observer-dependent. Quantitative analysis was added to compensate for these shortcomings. Third, a PET/CT image was taken by two different scanners, so that SUV_max_ and SUV_mean_ of the intestine were normalized with SUV_mean_ of the liver.

## Conclusions

To the best of our knowledge, this is the first report of the association between gut microbiota and physiologic bowel FDG activity. Our data demonstrated that the group with focal or intense FDG uptake in the intestine was associated with low abundance of unclassified Clostridiales. The group with intestinal FDG uptake lower than the liver was associated with high abundance of *Klebsiella*. We cautiously speculated that increased FDG activity might be caused by an increase in intestinal permeability and reflects impaired intestinal barrier function. Further studies with a larger sample is needed to confirm our speculation. If our hypothesis is true, it will help to determine the etiology of physiologic bowel FDG activity. Moreover, to replace a relatively invasive measurement, ^18^F-FDG PET/CT can provide a non-invasive method and contribute to widen the research field of intestinal permeability in the clinical or preclinical setting.

## Additional file

Additional file 1:
**Figure S1.** Comparisons of community alpha diversities among groups for the intestinal ^18^F-FDG. **Table S1.** Association between alpha diversity and intestinal ^18^F-FDG uptake. **Figure S2.** Principal coordinate analysis (PCoA) plot with Bray-Curtis distance. **Table S2-1.** Associations of intestinal ^18^F-FDG uptake with gut microbiota at the phylum level: Group 1 vs. 2. **Table S2-2**. Associations of intestinal ^18^F-FDG uptake with gut microbiota at the phylum level: Group 1 vs. 3. **Table S2-3.** Associations of intestinal ^18^F-FDG uptake with gut microbiota at the phylum level: Group 2 vs. 3. **Table S2-4.** Associations of intestinal ^18^F-FDG uptake with gut microbiota at the phylum level: Group 1 vs. 2 + 3. **Table S2-5.** Associations of intestinal ^18^F-FDG uptake with gut microbiota at the phylum level: Group 1 + 2 vs. 3. **Table S3.** Differing abundance of specific populations of gut microbiota between group 2 and group 3 for intestinal ^18^F-FDG uptake at the genus level. (PDF 1250 kb)

## Abbreviations

^18^F-FDG: Fluorine-18 fluorodeoxyglucose
BMI: Body mass index
CT: Computed tomography
FDR: False discovery rate
FODMAP: Fermentable oligo-, di- and mono-saccharides and polyols
GLM: Generalized linear model
GLUT: Glucose transporter
HbA1c: Hemoglobin A1c
HFD: High fat diet
log2FC: Log_2_ fold change
OTU: Operational taxonomic unit
PCoA: Principal coordinate analysis
PERMANOVA: Permutational multivariate analysis of variance
PET: Positron emission tomography
rRNA: Ribosomal Ribonucleic acid
SUV_max_: Maximum standardized uptake value
SUV_mean_: Mean standardized uptake value
TBR_max_: Uptake ratio of SUV_max_ of total bowel to SUV_mean_ of the liver
TBR_mean_: Uptake ratio of SUV_mean_ of total bowel to SUV_mean_ of the liver
VOI: Volume of interest
