# Supplementary material for: Gut microbiota and physiologic bowel 18F-FDG uptake
Source: EJNMMI Res. 2017 Aug 31;7:72. doi: 10.1186/s13550-017-0318-8 (PMC5578947; doi:10.1186/s13550-017-0318-8)
Supplement: Additional file 1: — Figure S1. Comparisons of community alpha diversities among groups for the intestinal 18F-FDG. Table S1. Association between alpha diversity and intestinal 18F-FDG uptake. Figure S2. Principal coordinate analysis (PCoA) plot with Bray-Curtis distance. Table S2-1. Associations of intestinal 18F-FDG uptake with gut microbiota at the phylum level: Group 1 vs. 2. Table S2-2. Associations of intestinal 18F-FDG uptake with gut microbiota at the phylum level: Group 1 vs. 3. Table S2-3. Associations of intestinal 18F-FDG uptake with gut microbiota at the phylum level: Group 2 vs. 3. Table S2-4. Associations of intestinal 18F-FDG uptake with gut microbiota at the phylum level: Group 1 vs. 2 + 3. Table S2-5. Associations of intestinal 18F-FDG uptake with gut microbiota at the phylum level: Group 1 + 2 vs. 3. Table S3. Differing abundance of specific populations of gut microbiota between group 2 and group 3 for intestinal 18F-FDG uptake at the genus level. (PDF 1250 kb) [file 13550_2017_318_MOESM1_ESM.pdf]

Supplementary Information

Gut microbiota and physiologic bowel ^{18}F-FDG uptake

Ji Yeon Kang[1§], Han-Na Kim[2§], Yoosoo Chang[3], Yeojun Yun[2], Seungho Ryu[3], Hocheol Shin[4*], and Hyung-Lae Kim[2*]

[1] *Department of Nuclear Medicine, Kangbuk Samsung Hospital, Sungkyunkwan University School of Medicine, Seoul, Korea*

[2] *Department of Biochemistry, Ewha Womans University, School of Medicine, Seoul, Korea*

[3] *Department of Occupational and Environmental Medicine, Kangbuk Samsung Hospital, Sungkyunkwan University School of Medicine, Seoul, Korea*

[4] *Department of Family Medicine, Kangbuk Samsung Hospital, Sungkyunkwan University School of Medicine, Seoul, Korea*

[§] These authors contributed equally to this work, and both should be considered as first author.

Corresponding authors: Hyung-Lae Kim, MD, PhD and Hocheol Shin, MD, PhD.

Address: (Hyung-Lae Kim) 1071, Anyangcheon-ro, Yangcheon-gu, 07985, Seoul, South Korea, Telephone: +82-2-2650-5727, E-mail: hyung@ewha.ac.kr; (Hocheol Shin) 29, Saemunan-ro, Jongnogu, 03181, Seoul, South Korea, Telephone: +82-2-2001-2012, E-mail: hcfm.shin@samsung.com

* These authors contributed equally to this work, and both should be considered as corresponding author.

Fig. S1. Comparisons of community alpha diversities among groups for the intestinal ^{18}F-FDG. Alpha diversity was measured by Chao1, ACE, Shannon, and InvSimpson indices between groups. There was no significant difference in ^{18}F-FDG uptake in all indices of alpha diversity between groups. The detailed summary statistics are shown in Tables S1.

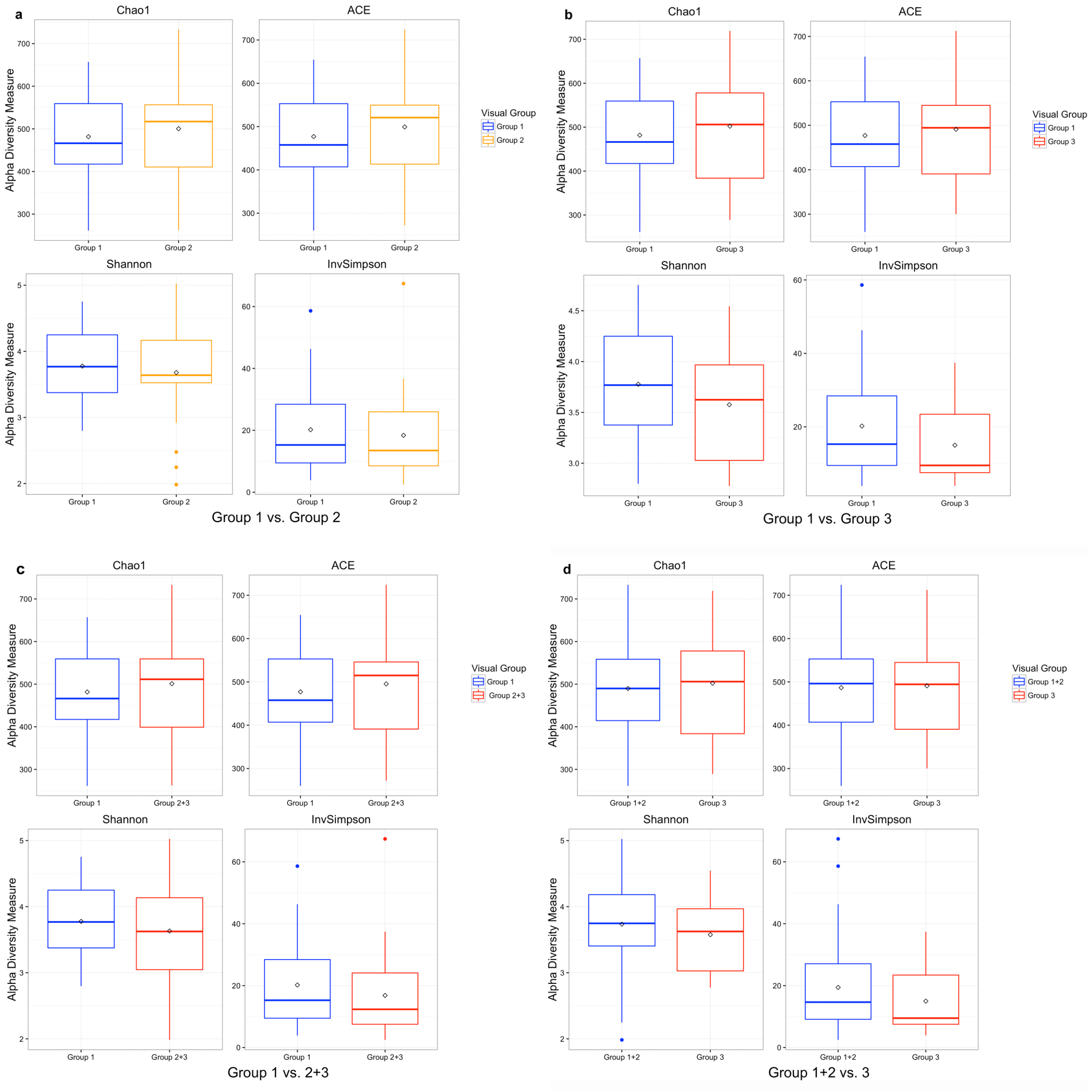

TableS1. Association between alpha diversity and intestinal ^{18}F-FDG uptake

		Chao1		ACE		Shannon		InvSimpson	
	groups	statistic	p.value	statistic	p.value	statistic	p.value	statistic	p.value
t-test	V_1vs2	-0.57	0.57	-0.68	0.50	0.47	0.64	0.41	0.68
	V_1vs3	-0.53	0.60	-0.37	0.72	1.10	0.28	1.38	0.18
	V_2vs3	-0.04	0.97	0.20	0.84	0.45	0.65	0.78	0.44
	V_1vs23	-0.69	0.49	-0.65	0.52	0.90	0.37	0.96	0.34
	V_12vs3	-0.33	0.74	-0.12	0.91	0.91	0.37	1.31	0.20
wilcox test	V_1vs2	233	0.56	234	0.58	267	0.89	286	0.58
	V_1vs3	198	0.58	209	0.78	261	0.33	278	0.16
	V_2vs3	175	0.89	186	0.64	190	0.56	187	0.62
	V_1vs23	431	0.49	443	0.60	528	0.52	564	0.25
	V_12vs3	373	0.79	395	0.96	451	0.36	465	0.26

Fig. S2. Principal coordinate analysis (PCoA) plot with Bray-Curtis distance. The PERMANOVA analysis showed that members of group 2 were significantly more dissimilar than them of group 1 (p=0.047, pseudo-F=1.57), although the comparison of the bacterial communities by PCoA plot could not easily distinguish the clear clustering between the two groups.

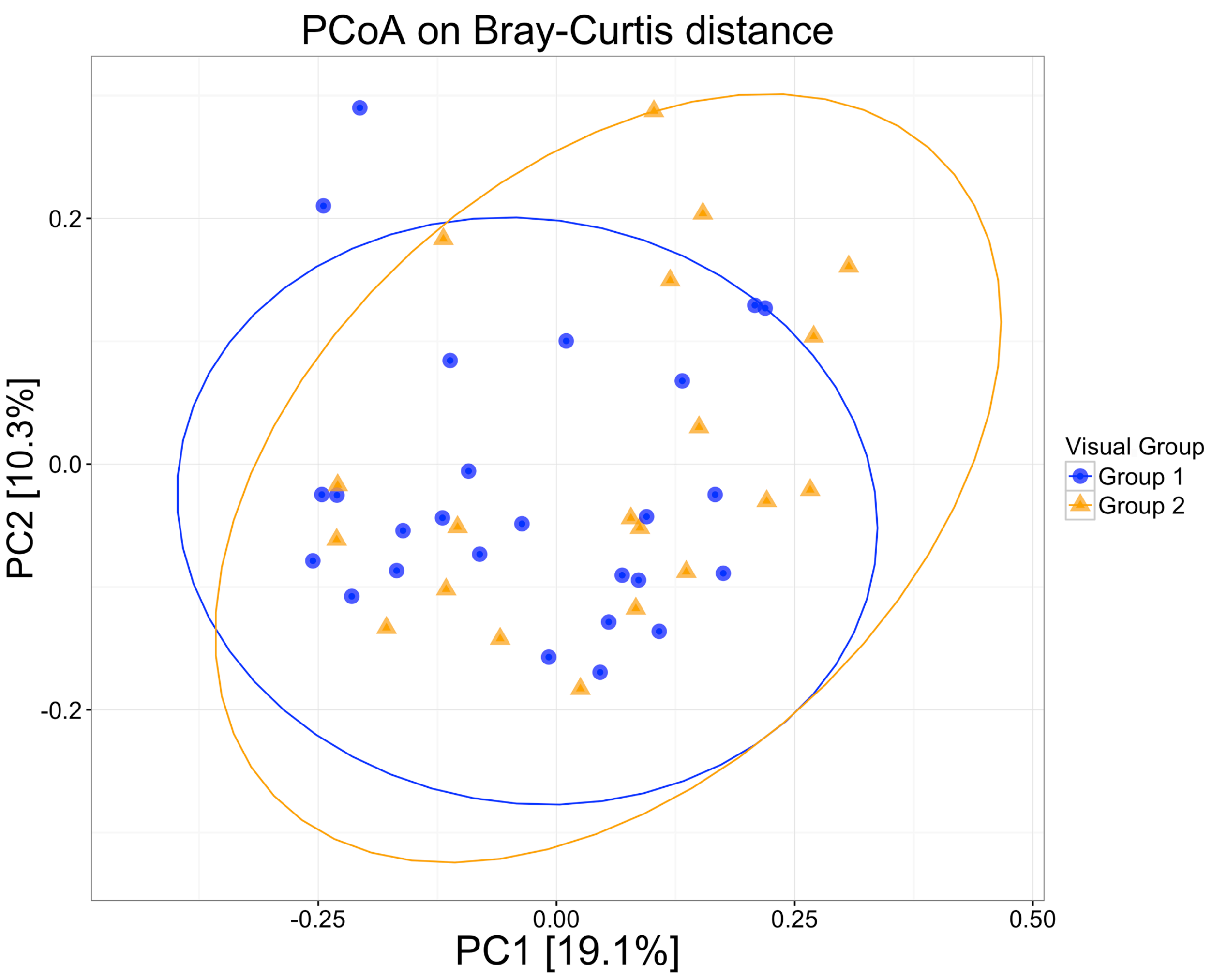

Table S2-1. Associations of intestinal ^{18}F-FDG uptake with gut microbiota at the phylum level: Group 1 vs. 2

Phylum	Relative Abundance in total samples (%)	baseMean	log2FoldChange (lfc)	lfcSE	stat	*p-value*	*q-value*
Proteobacteria	4.04	541.83	-0.77	0.43	-1.79	7.43.E-02	5.20.E-01
Bacteroidetes	34.40	9295.31	0.33	0.30	1.08	2.82.E-01	6.59.E-01
Verrucomicrobia	0.40	11.47	-0.69	0.60	-1.15	2.51.E-01	6.59.E-01
Actinobacteria	2.23	125.09	-0.02	0.43	-0.04	9.64.E-01	9.79.E-01
Firmicutes	58.63	5737.63	-0.07	0.20	-0.36	7.20.E-01	9.79.E-01
Fusobacteria	0.20	2.03	-0.10	0.58	-0.18	8.60.E-01	9.79.E-01
Tenericutes	0.27	21.57	0.02	0.61	0.03	9.79.E-01	9.79.E-01

Table S2-2. Associations of intestinal ^{18}F-FDG uptake with gut microbiota at the phylum level: Group 1 vs. 3

Phylum	Relative Abundance in total samples (%)	baseMean	log2FoldChange (lfc)	lfcSE	stat	*p-value*	*q-value*
**Fusobacteria**	0.20	225.50	4.50	1.40	3.21	1.33.E-03	**9.32.E-03**
Firmicutes	58.63	6264.39	-0.31	0.17	-1.84	6.57.E-02	2.30.E-01
Bacteroidetes	34.40	11282.56	0.30	0.30	0.98	3.27.E-01	4.00.E-01
Proteobacteria	4.04	773.70	-0.73	0.57	-1.28	1.99.E-01	4.00.E-01
Tenericutes	0.27	18.31	-1.15	1.21	-0.95	3.43.E-01	4.00.E-01
Verrucomicrobia	0.14	42.11	1.66	1.41	1.18	2.39.E-01	4.00.E-01
Actinobacteria	2.23	165.08	0.40	0.58	0.70	4.87.E-01	4.87.E-01

Table S2-3. Associations of intestinal ^{18}F-FDG uptake with gut microbiota at the phylum level: Group 2 vs. 3

Phylum	Relative Abundance in total samples (%)	baseMean	log2FoldChange (lfc)	lfcSE	stat	*p-value*	*q-value*
Proteobacteria	4.04	487.12	0.85	0.51	1.67	0.09	0.38
Firmicutes	58.63	7499.91	-0.29	0.21	-1.39	0.17	0.44
Bacteroidetes	34.40	14258.32	0.10	0.39	0.26	0.80	0.84
Verrucomicrobia	0.40	51.12	-0.62	3.10	-0.20	0.84	0.84
Tenericutes	0.27	21.38	-0.95	2.98	-0.32	0.75	0.84
Fusobacteria	0.20	309.91	1.81	3.09	0.58	0.56	0.84
Actinobacteria	2.23	209.61	0.43	0.67	0.64	0.52	0.84

Table S2-4. Associations of intestinal ^{18}F-FDG uptake with gut microbiota at the phylum level: Group 1 vs. 2+3

Phylum	Relative Abundance in total samples (%)	baseMean	log2FoldChange (lfc)	lfcSE	stat	*p-value*	*q-value*
Actinobacteria	2.23	144.88	0.19	0.52	0.37	7.E-01	7.E-01
Bacteroidetes	34.40	10447.66	0.30	0.27	1.13	3.E-01	7.E-01
Firmicutes	58.63	5860.97	-0.15	0.16	-0.98	3.E-01	7.E-01
Fusobacteria	0.20	145.88	1.11	0.96	1.16	2.E-01	7.E-01
Proteobacteria	4.04	572.31	-0.40	0.96	-0.41	7.E-01	7.E-01
Tenericutes	0.27	18.54	-0.40	0.85	-0.47	6.E-01	7.E-01
Verrucomicrobia	0.14	28.13	0.70	1.01	0.69	5.E-01	7.E-01

Table S2-5. Associations of intestinal 18F-FDG uptake with gut microbiota at the phylum level: Group 1+2 vs. 3

Phylum	Relative Abundance in total samples (%)	baseMean	log2FoldChange (lfc)	lfcSE	stat	*p-value*	*q-value*
Fusobacteria	0.20	145.88	3.59	1.50	2.40	1.65.E-02	1.16.E-01
Firmicutes	58.63	5860.97	-0.28	0.17	-1.63	1.02.E-01	2.77.E-01
Verrucomicrobia	0.14	28.13	2.07	1.33	1.56	1.19.E-01	2.77.E-01
Tenericutes	0.27	18.54	-1.33	1.03	-1.29	1.99.E-01	3.48.E-01
Actinobacteria	2.23	144.88	0.41	0.58	0.70	4.86.E-01	6.65.E-01
Bacteroidetes	34.40	10447.66	0.17	0.30	0.57	5.70.E-01	6.65.E-01
Proteobacteria	4.04	572.31	-0.07	1.50	-0.05	9.63.E-01	9.63.E-01

Table S3. Differing abundance of specific populations of gut microbiota between group 2 and group 3 for intestinal ^{18}F-FDG uptake at the genus level

Group	Genus	Relative Abundance (%)[a]	Log2FoldChange (SE)	*p-value*	*q-value*
2 vs. 3	Unclassified Clostridiales	4.10	-1.15 (0.38)	0.003	0.18
	Holdemania	0.06	-1.94 (0.87)	0.026	0.65
	Unclassified_Ruminococcaceae	13.56	-0.51 (0.27)	0.064	0.65
	Unclassified_Mogibacteriaceae	0.30	-1.16 (0.65)	0.072	0.65
	Unclassified_S24-7	0.53	2.28 (1.33)	0.086	0.65

[a]Relative abundance in total 63 samples

The top 5 ranked taxa were shown because there is no statistically significant difference between group 2 and group 3.
